# Cerebrovascular Impedance as a Function of Cerebral Perfusion Pressure

**DOI:** 10.1109/OJEMB.2023.3236267

**Published:** 2023-01-12

**Authors:** Jason Yang, Deepshikha Acharya, William B. Scammon, Samantha Schmitt, Emily C. Crane, Matthew A. Smith, Jana M. Kainerstorfer

**Affiliations:** Department of Biomedical EngineeringCarnegie Mellon University 6612 Pittsburgh PA 15213 USA; Department of Biomedical EngineeringCarnegie Mellon University 6612 Pittsburgh PA 15213 USA; Neuroscience InstituteCarnegie Mellon University 6612 Pittsburgh PA 15213 USA

**Keywords:** Cerebral autoregulation, cerebral hemodynamics, cerebrovascular impedance, diffuse correlation spectroscopy, near-infrared spectroscopy

## Abstract

*Goal:* Cerebrovascular impedance is modulated by a vasoactive autoregulative mechanism in response to changes in cerebral perfusion pressure. Characterization of impedance and the limits of autoregulation are important biomarkers of cerebral health. We developed a method to quantify impedance based on the spectral content of cerebral blood flow and volume at the cardiac frequency, measured with diffuse optical methods. *Methods:* In three non-human primates, we modulated cerebral perfusion pressure beyond the limits of autoregulation. Cerebral blood flow and volume were measured with diffuse correlation spectroscopy and near-infrared spectroscopy, respectively. *Results:* We show that impedance can be used to identify the lower and upper limits of autoregulation. *Conclusions:* This impedance method may be an alternative method to measure autoregulation and a way of assessing cerebral health non-invasively at the clinical bedside.

## I. Introduction

Cerebral autoregulation (CA) is the brain's compensation mechanism to maintain a constant cerebral blood flow (CBF). This CA mechanism modulates cerebrovascular impedance (CVI) in response to changes in cerebral perfusion pressure (CPP). Changes in CVI are achieved through active vasoconstriction and vasodilation in response to slow baseline fluctuations in CPP, such as those induced by exercise or postural changes [1], [2], [3]. However, CA function can be disrupted in many disease states such as stroke [4], [5], [6], [7], traumatic brain injury [7], [8], and hydrocephalus. We have recently introduced a novel way of estimating CVI and thus CA. Here we experimentally validate this approach in non-human primates.

The autoregulatory function can be characterized by measuring the CBF response to changes in CPP or arterial blood pressure (ABP). For slow and steady-state conditions, Lassen's curve defines a range of CPP values where CA is intact and CBF remains near-constant [1], [9], [10], [11]. Outside this autoregulatory range, CBF rises and falls with CPP. For dynamic changes in CBF, CA was found only to be active at slow (∼< 0.1 Hz) changes in ABP [1], [12], [13], [14], [15], [16]. At higher frequencies, such as cardiac pulsations, no active vasomotor response, thus CA, is observed and changes in CPP and CBF are related solely through CVI.

The CPP limits of CA can be assessed by Lassen's curve or dynamic slow changes in CBF [1], [3]. Here we propose to use CVI to evaluate CA limits as an alternative to Lassen's curve. For this, we use the cardiac pulsation frequency, which does not trigger a vasomotor response. We propose CVI as a function of CPP as an indirect marker of CA function [1], [9], [11].

Impedance is the frequency-dependent ratio of spectral pressure and spectral flow. Like resistance, which is commonly referred to in CA literature [1], [3], impedance relates a vessel's pressure and flow dynamics [17], [18]. However, calculating vascular resistance (the time-averaged component of pressure divided by flow) is only valid at a steady state. By contrast, impedance can be calculated for cardiac frequencies and can account for physiological parameters such as vessel compliance, blood inertia, or other frequency-dependent quantities that would otherwise confound resistance calculations [18], [19].

Estimation of impedance requires estimations of blood pressure and flow at a sufficiently high temporal resolution to recover information at the cardiac frequencies and harmonics. Moreover, pressure and flow estimations should be from similar vascular compartments to account for different pressure environments. Such measurements have been achieved before by using TCD combined with pulsatile blood pressure from the finger [19] or femoral artery [17], [18], [20], but these methods are not bedside compatible for prolonged monitoring. Techniques such as diffuse correlation spectroscopy (DCS) and near-infrared spectroscopy (NIRS) are bedside compatible, allow for prolonged monitoring, and can measure co-localized pulsatile blood flow and blood volume, respectively, with high temporal resolution.

In this work, we will present a method that utilizes a custom high-speed DCS-NIRS device capable of measuring CVI changes using cardiac signals. We will show results from three acute studies using non-human primates (NHPs) and demonstrate the feasibility of using CVI as a measure of CA function in comparison with Lassen's curve. This novel method may provide a way to assess cerebral health at the clinical bedside and will be beneficial in disease monitoring where CA is known to be impaired.

## II. Materials and Methods

We designed a combined DCS-NIRS device to measure co-localized pulsatile blood flow and hemoglobin concentration changes. We recorded pulsatile CBF changes and blood volume changes in three animals at different CPP levels. CVI was calculated based on the spectral ratio of blood volume and flow.

### A. Combined DCS-NIRS Device

**Fig. 1.**
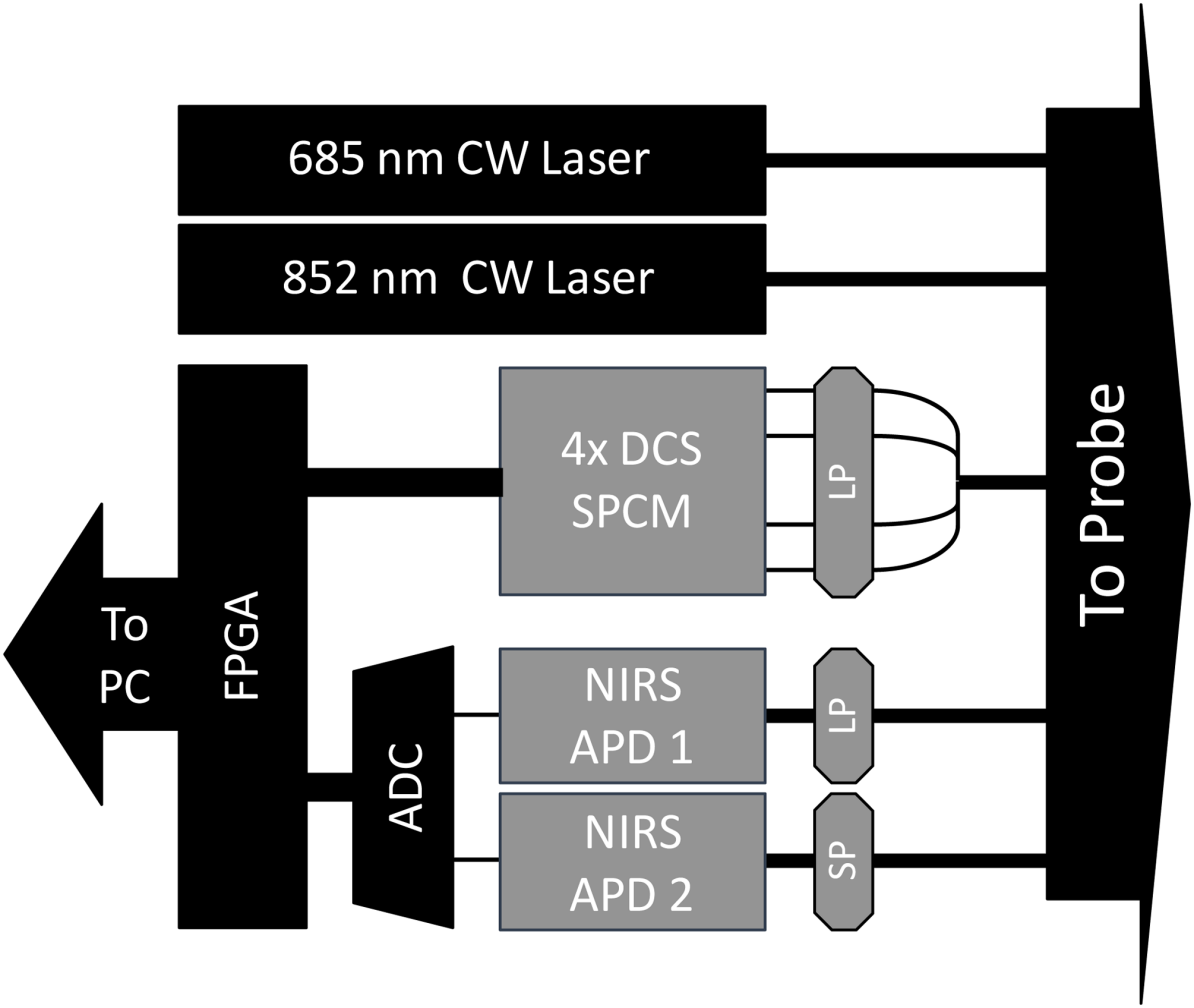
Schematic of combined DCS-NIRS imaging device showing two continuous wave (CW) laser sources, four SPCMs for DCS, and two APDs for NIRS. The detectors have long-pass (LP) or short-pass (SP) filters to be sensitive to a single wavelength. An FPGA is used to synchronize and collect data from the detectors and forwarded to a computer for further processing.

We designed both DCS and NIRS subsystems in the same compact enclosure sharing the same laser sources for illumination. All detectors were controlled using the same field programmable gate array (FPGA) (XC7A100T-2CSG324C, Xilinx, San Jose, CA) and sampled using the same clock source to ensure synchronization between the subsystems. DCS and NIRS data were interleaved together and sent to a computer for further processing. Fig. 1 shows the high-level device schematic.

We used DCS to measure changes in CBF. The operating principles of DCS have been described previously [21], [22], [23], [24], [25]. A long coherence length laser operating at λ = 852 nm at 35 mW (IBEAM-SMART-852-S-WS, Toptica Photonics, Germany) was used for illumination through a single mode fiber. The source-detector distance was 15 mm and photons were collected using a co-localized bundle of four few-mode fibers leading to an array of four high-speed single-photon counting modules (SPCM) (SPCM-AQ4C, Excelitas, Canada). The detector fiber ends were coated with a long-pass filter (λ_c_ = 800 nm) to block extraneous interfering light sources. Photon arrival times were recorded and binned in 500 ns wide windows. Intensity autocorrelation curves for each of the four SPCM detectors were calculated in software over 20 ms and then averaged together resulting in a signal bandwidth of 50 Hz. This process was repeated every 2 ms, resulting in a 500 Hz sample rate. We then calculated the instantaneous intensity autocorrelation signal-to-noise ratio (SNR) curve for each point using the equation:

\begin{equation*}
\text{SNR}\left( {\rm{\tau }} \right) = \frac{{\overline {{g}_2\left( {\rm{\tau }} \right)} - 1}}{{{\sigma }_{{g}_2}\left( {\rm{\tau }} \right)}}
\end{equation*}

where $\overline {{g}_2( {\rm{\tau }} )} $ is the mean temporal autocorrelation and ${{\rm{\sigma }}}_{{g}_2}( {\rm{\tau }} )$ is the standard deviation per delay time over the previous 10 s of data [25]. We extracted the CBF-related parameter $\alpha Db$ and the speckle contrast parameter β using a multi-parameter fit to the analytical correlation-diffusion equation [21], [25], [26]. This fit was weighted by the SNR curve to minimize the effect of instrument and quantization noise on the fitting function.

**Fig. 2.**
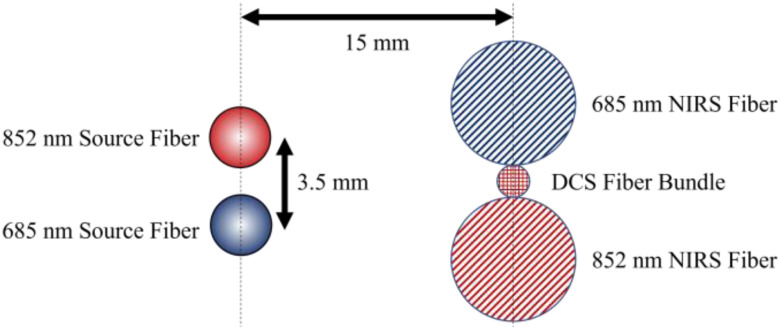
Optical probe design showing the relative locations and spacing of the source fibers (left) and the detector fibers (right). The red and blue colors denote the 852 nm and 685 nm carrying fibers, respectively.

The CW-NIRS subsystem used a second fiber-coupled laser operating at 18 mW, λ = 685 nm (IBEAM-SMART-685-S-WS, Toptica Photonics, Germany) in addition to the 852 nm laser. We placed two 800 μm fibers tangential to the DCS detector fiber, 15 mm away from the laser source, leading to two linear avalanche photodiodes (APD) detectors (APD440A, Thorlabs). Fig. 2 shows the arrangement of the DCS and NIRS fibers in the optical probe. A long or short pass filter (FF01-685 and FF01-842, Semrock, Rochester, NY) was placed in front of each detector for wavelength differentiation. Detector intensity information was recorded using a 14-bit ADC at 500 ksps, then averaged and decimated to 500 Hz in software. A 50 Hz low-pass filter was applied during decimation to prevent aliasing. We calculated Δμ_a_ using the modified Beer-Lambert law to derive oxy (ΔHbO), deoxy (ΔHb), and total (ΔHbT = ΔHbO + ΔHb) hemoglobin concentration changes [27].

### B. Experimental Design

All procedures were approved by the Institutional Animal Care and Use Committee of Carnegie Mellon University under IACUC# PROTO202000007 on 19 Feb 2021 and complied with guidelines stated in the National Institute of Health's *Guide for the Care and Use of Laboratory Animals* (2011). Measurements were taken on cynomolgus macaques (*Macaca fascicularus,* N = 3, two females and one male). All animals were initially sedated using 20 mg/kg of ketamine with 0.01 mg/kg of Glycopyrrolate. After intubation, animals were ventilated and maintained under anesthesia using a combination of 10–25 μg/kg/hr of intravenous fentanyl and a minimal amount of isoflurane gas (0.1 – 1.0 %). Animals were then paralyzed using 0.1 mg/kg/hr of vecuronium bromide.

The combined DCS-NIRS probes were placed over the right parietal lobe, directly on the exposed skull to minimize the influence of the scalp and muscle layers on the optical recording. We measured continuous ICP using a pressure probe (NEUROVENT-P, Raumedic, Germany) placed in the brain parenchyma through a cranial burr hole sealed with bone wax. We measured ABP via a pressure transducer (Smith Medical TransStar, Dublin, OH) connected to an arterial line placed in the external carotid artery. Both ICP and ABP signals were measured continuously at 100 Hz (MPR1 Datalogger, Raumedic, Germany). The MPR1 and the combined DCS-NIRS system were synchronized through alignment voltage pulses sent to both devices simultaneously. We placed a catheter through a second cranial burr hole into the lateral ventricle and sealed it with bone wax, to allow for manipulation of ICP. This ventricular catheter was connected to a saline gravity reservoir that could be raised relative to the animal's head to induce ICP changes. To induce ABP changes, we infused each animal with 1.05 - 5.60 μg/kg/min of phenylephrine via a constant infusion pump, where the rate of infusion was altered to change the ABP.

**Fig. 3.**
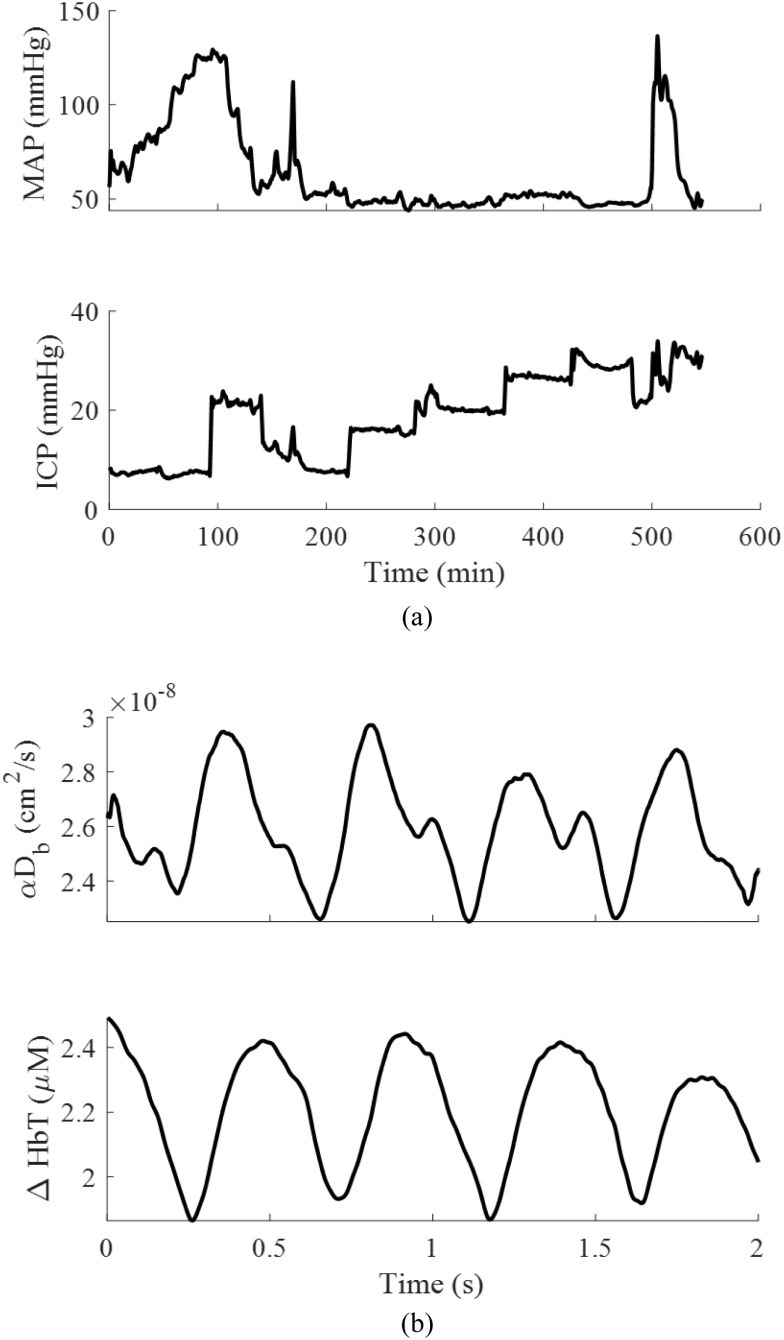
Example recording from one subject. (a) ABP and ICP low pass filtered at 0.01 Hz to show baseline drifts. (b) A 2 second window of CBF and ΔHbT showing individual cardiac pulses. The pulses were filtered using a 0.1 second moving average.

A 30-minute baseline measurement was recorded at the opening ICP and ABP after which we modulated ABP by infusing the animal with phenylephrine in steps of 1.05 μg/kg/min for 10 min per step. We then flushed the phenylephrine infusion line and replaced it with saline until the animals returned to a steady state baseline. After that, we raised the ventricular saline reservoir to induce increasing steps of 5 mmHg in ICP, up to 30 - 40 mmHg. Each ICP level was held for approximately 60 min. At the highest ICP level, phenylephrine was infused at 5.60 μg/kg/min again to raise ABP. Time traces are seen in Fig. 3.

### C. Signal Processing

We used Matlab R2021b (The MathWorks Inc., Natick, MA, USA) for signal processing. For impedance calculations, cardiac pulsation frequencies were isolated by first mean subtracting and then applying a high pass filter $( { {f}_c = \text{1.2 Hz}} )$. We assumed that the blood vessels had a constant elastic modulus [28], [29] thus pulsatile CBV changes could be used as a surrogate measurement of pulsatile blood pressure changes. We segmented CBF and CBV changes into 30 s analysis windows with a 15 s overlap, and a fast Fourier transform (FFT) was computed to calculate the CBF and CBV spectra. We used Matlab's *findpeaks* function to determine the index of the cardiac fundamental frequency and first harmonic in the CBF and CBV spectra. A signal-noise ratio (SNR) for each window was calculated as the ratio of the CBF and CBV spectral power at the fundamental frequency to the power in between the fundamental and harmonic. We rejected any window where the CBF or CBV SNR was less than 3. Impedance can be calculated as

\begin{equation*}
Z\left( {j\omega } \right) = \frac{{P\left( {j\omega } \right)}}{{Q\left( {j\omega } \right)}}
\end{equation*}

where *P* and *Q* represent the frequency (*j*ω) dependent pressure and flow, respectively. Here, $Q = {\rm{\Delta \alpha }}{D}_b = {\rm{\Delta CBF}}$. With the assumption of constant elastic modulus for vessels, pulsatile pressure changes are proportional to pulsatile blood volume changes, and thus ${\rm{\Delta }}P = {\rm{\Delta HbT}} = {\rm{\Delta CBV}}$ [29]. We computed an impedance index for every window by summing together the magnitude of the impedance spectra at the fundamental and the first harmonic frequencies. Additionally, the mean CPP, ABP, ICP, and CBF were calculated for each analysis window. Because of the availability of pulsatile ABP measured from the carotid and ICP in the parenchyma, we calculated an additional impedance index where ${\rm{\Delta }}P = {\rm{\Delta CPP}}$ and $Q = {\rm{\Delta }}CBF$ for each window. CPP was calculated as the difference between ABP and ICP.

Autoregulative impedance index curves for each subject were calculated by taking the computed impedance index for each analysis window and binning by CPP in 3 mmHg wide bins. Similarly, we calculated Lassen's curves by binning the average CBF in each analysis window. Any bin with one or fewer samples was discarded. To determine a cohort average, the individual impedance index and Lassen's curves were normalized to the CPP = 65 mmHg bin and averaged over all animals.

## III. Results

We successfully recorded pulsatile CBF and changes in total hemoglobin (ΔHbT) for multiple hours using the DCS-NIRS device in three NHPs. We modulated MAP and intracranial (ICP) using phenylephrine IV infusions and fluid induction, respectively. Using diffuse optical measurements, in addition to invasive pressure probes, we reconstructed Lassen's autoregulation curve. Additionally, by analyzing the spectral information in the cardiac waveforms, we generated an impedance index curve.

### A. Hemodynamic Modulations

For the three animals, the baseline MAP was 69 ± 10 mmHg and reached a maximum of 134 ± 10 mmHg during phenylephrine administration. After flushing the phenylephrine lines with saline, the animals returned to near-baseline MAP of 67 ± 11 mmHg. Fig. 3(a) shows data from one animal with a 30-minute baseline, phenylephrine-driven stepwise increases in MAP, and stepwise increases in ICP. Because of the high temporal resolution of our combined DCS-NIRS device, we can observe individual cardiac pulses in both ΔCBF and ΔHbT (Fig. 3(b)).

### B. Lassen's and Impedance Index Curves

**Fig. 4.**
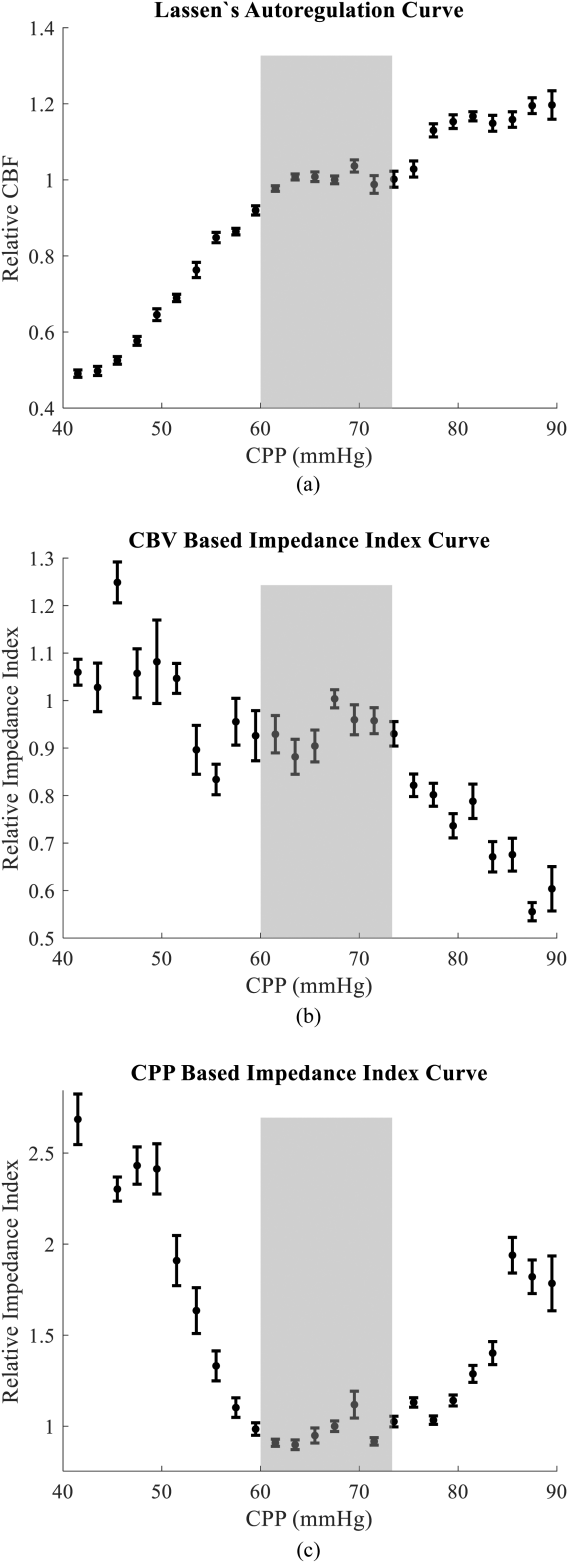
(a) Group averaged Lassen's curve showing a plateau of intact autoregulation from 60 – 72 mmHg. (b) The CBV-based autoregulative impedance index curve showing the positive relationship between impedance and CPP within the autoregulative limits. (c) The CPP-based autoregulative impedance index curve. The shaded areas represent regions of intact autoregulation. Error bars represent standard error from the mean.

Fig. 4 shows the group averaged Lassen's as well as the CBV and CPP-based impedance index curves. Data below CPP = 40 mmHg was rejected due to the SNR metric. Data above CPP = 90 mmHg could not be induced reliably across all animals and were similarly truncated. Error bars represent standard error from the mean. Lassen's curve in Fig. 4(a) shows an intact autoregulative plateau between approximately 60 – 72 mmHg. Beyond these limits, CBF rises and falls passively with CPP. Fig. 4(b) shows the CBV-based impedance index vs. CPP curve where the impedance increases with CPP within the upper limit of autoregulation (ULA) and lower limit of autoregulation (LLA) defined in Fig. 4(a). Outside these limits, the autoregulatory capacity is exhausted and the impedance index shows a negative dependence with CPP. Fig 4(c) shows the CPP-based impedance index vs. CPP. Here, like in the CBV-based curves, the impedance index shows a negative dependence on CPP below the LLA but deviates from CBV trends above the ULA.

## IV. Discussion

In this work, we demonstrated the use of a novel design for an integrated DCS-NIRS device to record high temporal resolution pulsatile CBF and CBV changes. This allows us to estimate CVI as a metric for CA in three animals. In our experiment, wherein we modulated CPP by varying ICP and MAP to challenge cerebral autoregulation, we were able to reconstruct an impedance index curve that resembles resistance curves shown in the literature [3], [30]. These impedance index curves could provide an alternative method to assess the lower and upper limits of CA.

### A. Novel Combined DCS-NIRS

We developed a combined DCS-NIRS device that shares a single laser source without crosstalk or clock synchronization issues that would typically occur using separate instruments. Our device allowed for the co-localization of the NIRS and DCS optical signals with sufficient temporal bandwidth and noise to capture the hemodynamic cardiac frequencies and harmonics. Because our method relies only on changes in impedance, not absolute values, the higher sample rate, and relativistic measurements provided by CW-NIRS were sufficient. Other DCS-NIRS implementations using CW-NIRS techniques have relied on shutter systems to time-interleave NIRS and DCS acquisition such that light sources from the respective subsystems do not interfere with data collection from their counterparts [31]. However, this method can limit acquisition rates and does not allow for signal oversampling. By using a filter-based approach in our combined DCS-NIRS device, we were able to achieve the high-fidelity and high temporal resolution ΔCBF and ΔCBV measurements necessary for impedance analysis.

### B. Estimation of Pulsatility

In our work, we required a way of measuring pulsatile pressure changes in the same vascular bed as our DCS-based flow measurements. By co-localizing our NIRS system and assuming a constant elastic modulus vascular bed during a cardiac pulsation [28], [29], we used ΔCBV as a surrogate measurement of blood pressure pulsatility. For the results presented, we assumed that the pressure-volume relationship was monotonic and linear. However, the pressure environment in the cerebral microvasculature may present a non-linear pressure-volume relationship. To account for such non-linearity, we also assumed an exponential pressure-volume relationship. We found (data not shown) that the impedance index autoregulative curves still follow the trends shown in Fig 4(c).

### C. Impedance and Autoregulation

In this study, we calculated CVI at the cardiac frequencies. When perturbing autoregulation by changing CPP, we found that the CBV-based impedance index using co-localized NIRS and DCS may be used to determine the upper and lower limits of CA much like the canonical Lassen's curve. Because of the similarities between resistance and impedance, our work is consistent with models describing the relationship between CVR and CPP [3], [30] which shows that autoregulative-driven vasoconstriction causes an increase in resistance. Outside these limits, the vasculature's autoregulative ability is exhausted and the vessels can no longer vasoconstrict or vasodilate any further [4], [7], [32], [33]. Above the upper limit of autoregulation, cerebral vessels dilate due to pressure resulting in a decrease in impedance. Below the lower limit, vessels undergo partial collapse [32], [34]. Overall, the limits of autoregulation captured by the CBV-based impedance index show good agreement with the limits defined by Lassen's curve.

The CPP-based impedance index showed similar trends to the CBV-based impedance index below the LLA. However, the CPP-based impedance differed from the CBV-based impedance above the ULA. This discrepancy could be due to the pressure signal originating, in part, from outside the skull. Because the impedance environment inside and outside the skull differs, the pressure waveform within the skull where CBF is being measured with DCS may be different. Without accounting for the changes in the waveform shape due to differences in location, impedance cannot be reliably calculated in this manner. Because the signals used for the CBV-based impedance index are co-localized with CBF, we believe the CBV-based calculation to be a more accurate representation of impedance.

### D. Impedance vs. Resistance

The use of CVI to estimate the limits of autoregulation has some practical advantages over CVR. Notably, CVR requires measurements of steady-state pressure and flow. Due to natural fluctuations, such as respiration and low-frequency changes [1], [11], [16] steady-state is at best an approximation. The CVI calculation introduced here does not make such assumptions. In addition, experiments that have explored CVR typically have relied on measuring pressure and flow at two locations of the body and correcting for positional changes which might limit the accuracy of the measurement [2]. By measuring impedance and focusing on the cardiac fluctuations in the hemodynamic signals, we can measure co-locally as well as potentially extract frequency-dependent information about vessel compliance and fluid inertia that is lost in simple resistance measurements [18], [19]. Moreover, by accounting for these frequency-dependent parameters, CVI measurements can be sampled very quickly, whereas CVR measurements may require longer time averaging to ensure a steady-state measurement.

### E. Limitations and Future Work

We have shown that CVI can be used to determine the CPP limits of CA intactness. One limitation of this method is the reliance on measurements of CPP, and thus ICP, which necessitates the use of invasive pressure probes [35], [36]. However, previous work has been done to estimate ICP non-invasively using DCS [8], [37]. Combining these two techniques may allow for a completely non-invasive autoregulation assessment in the future.

Additionally, determining the LLA and ULA using this method relies on modulating CPP to reconstruct the impedance autoregulative index curve. This may limit this method since wide CPP changes in critically ill patients may not be well tolerated. However, this method may still be used to provide a binary measure if a subject is autoregulating or not. If an increase in impedance is observed with a slight increase in CPP, then the patient may be within the LLA and ULA. Conversely, if impedance decreases with a small increase in CPP, the patient may be outside the limits.

Finally, our approach is also limited to relative changes in CVI. Our approach is limited to relative CBV and thus relative pulsatile pressure and relative CBF changes. To quantify absolute impedance, a measurement of absolute changes in flow and pressure would be required.

## V. Conclusion

We have demonstrated the potential for DCS and NIRS to be used to estimate relative CVI changes. By combining changes in pulsatile CBV as a surrogate measurement for pulsatile pressure changes, combined with pulsatile CBF, we have shown that an impedance index can be estimated by taking the spectral ratio of pressure and flow at the cardiac frequencies and harmonics. When plotted as a function of CPP, the impedance index shows a positive trend within the limits of CA defined by Lassen's curve. This is consistent with autoregulation-driven changes in cerebrovascular resistance.

Overall, the measurement of changes in CVI can be another metric to assess CA and may serve as a biomarker of cerebral health.
